# Case Report: Awake double-lumen tube intubation and intraoperative ventilation strategy conversion in an obese adolescent with a giant anterior mediastinal teratoma

**DOI:** 10.3389/fmed.2026.1882179

**Published:** 2026-08-07

**Authors:** Kun Wang, Li Su, Gang Li

**Affiliations:** 1 Department of Anesthesiology, Beijing Haidian Hospital, Beijing, China; 2 Department of Anesthesiology, Peking University Third Hospital, Beijing, China

**Keywords:** adolescent, anterior mediastinal mass, awake intubation, case report, double-lumen tube, obesity, permissive hypercapnia, spontaneous ventilation

## Abstract

**Background:**

Anterior mediastinal masses may precipitate life-threatening airway or cardiovascular collapse during anesthesia. In obese adolescents, symptom-based assessment may underestimate the risk when substantial airway compression and pericardial effusion are present.

**Case presentation:**

A 13-year-old boy with class II obesity (body mass index [BMI] 36.0 kg/m^2^) presented with a 10.4 × 9.7 cm anterior mediastinal mass compressing both the trachea and the left main bronchus, accompanied by a moderate-to-large pericardial effusion. Despite minimal respiratory symptoms, imaging findings supported a high-risk classification. Awake oral intubation using a 37 Fr left double-lumen tube (DLT) was performed without sedation under multilevel topical anesthesia while spontaneous ventilation was maintained. Fiberoptic bronchoscopy revealed a slit-like left main bronchial orifice, and the bronchial tip could not be advanced safely. The DLT was withdrawn into the main airway, and both lumens were used for bilateral ventilation. Transient permissive hypercapnia occurred (PaCO₂ 52.7 mmHg, pH 7.289) with preserved oxygenation, and PaCO₂ improved after thoracotomy without changes in ventilator settings. The tumor was completely resected, and pathology confirmed a mature teratoma.

**Conclusion:**

This case illustrates a practical airway-first strategy for managing a high-risk adolescent with an anterior mediastinal mass and suggests that, in selected patients with an already secured main airway, temporary bilateral ventilation through both DLT lumens may serve as a feasible rescue option when bronchial positioning fails.

## Introduction

Anterior mediastinal masses may compress the tracheobronchial tree, heart, and great vessels. Loss of airway tone, changes in transpleural pressure, positive-pressure ventilation, and neuromuscular blockade can convert a compensated preoperative state into acute airway obstruction or hemodynamic collapse (1–5). Pediatric and adolescent patients may be particularly vulnerable because smaller airway caliber and greater airway compliance can magnify the physiological effects of extrinsic compression (1, 4, 5).

Risk assessment commonly integrates symptoms, airway imaging, vascular or cardiac compression, and positional tolerance (1–7). However, the relationship between symptoms and anatomical findings is imperfect. Obesity can reduce functional residual capacity and alter respiratory mechanics (8, 9), which may complicate clinical interpretation when substantial airway compression is present despite limited symptoms. Awake intubation with preserved spontaneous ventilation is a recognized strategy for anticipated difficult or high-risk airways (10); however, for thoracic surgery requiring lung isolation, placement of a DLT can be difficult or hazardous when the tracheobronchial tree is compressed (11, 12). Reports describing the use of a DLT during mediastinal mass surgery have primarily emphasized airway stenting or lung isolation (13, 14), whereas less has been written about the physiological consequences of retaining the DLT in the main airway for bilateral ventilation when bronchial positioning fails.

We report such a case in an obese adolescent with a giant anterior mediastinal teratoma and discuss the decision-making and ventilatory observations encountered during the perioperative period.

## Case description

### Patient information and preoperative findings

A 13-year-old boy (height 178 cm, weight 114 kg, BMI 36.0 kg/m^2^) was admitted with a 4-month history of chest tightness and chest pain, along with a worsening cough over the preceding month. He had no known medical history or drug allergies.

Physical examination revealed a midline trachea without jugular venous distension, facial edema, stridor, orthopnea, or exertional dyspnea. Peripheral oxygen saturation was 97–98% in both the supine and semi-recumbent positions, and no positional hemodynamic changes were observed.

**Figure 1:**
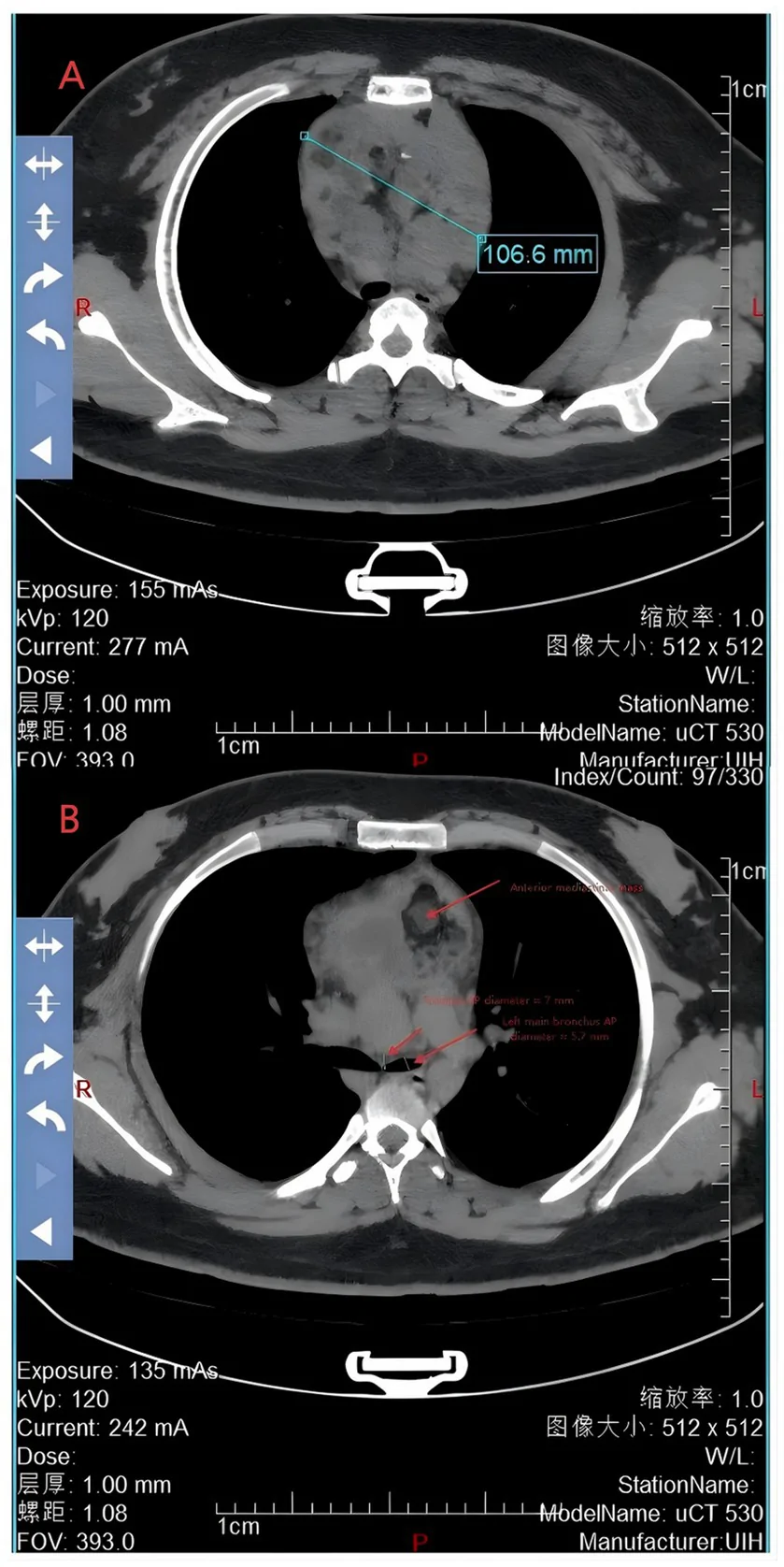
Preoperative contrast-enhanced chest CT (mediastinal window). **(A)** Axial image at the level of maximum tumor cross-section showing a 10.4 × 9.7 cm heterogeneous anterior mediastinal mass with mixed soft-tissue, fluid, calcified, and fat-density components. The mass displaced the heart to the left. **(B)** Axial image at the carina level demonstrating posterior compression of the trachea (anteroposterior diameter ≈ 7 mm, ~50% stenosis) and marked narrowing of the left main bronchus (anteroposterior lumen ≈ 5.7 mm, ~55% stenosis). The carina is deformed and displaced.

Contrast-enhanced chest computed tomography (Figure 1) demonstrated a 10.4 × 9.7 cm heterogeneous anterior mediastinal mass containing soft-tissue, fluid, calcified, and fat-density components. The mass compressed the trachea posteriorly (anteroposterior diameter approximately 7 mm, representing approximately 50% stenosis) and narrowed the left main bronchus (anteroposterior lumen approximately 5.7 mm, representing approximately 55% stenosis), while displacing the heart to the left. No obvious superior vena cava or pulmonary artery compression was identified. Transthoracic echocardiography demonstrated moderate-to-large pericardial effusion with a maximum depth of 16 mm, no right-chamber diastolic collapse, an inferior vena cava diameter of 21 mm with diminished respiratory variability, mild tricuspid regurgitation, and a left ventricular ejection fraction of 63%. Room-air arterial blood gas analysis showed a pH of 7.391, PaCO₂ of 37.6 mmHg, PaO₂ of 88.0 mmHg, and a lactate level of 2.2 mmol/L.

The principal differential diagnoses for an anterior mediastinal mass in an adolescent include lymphoma, germ cell tumors, including mature or immature teratoma, thymic lesions, lymphangioma, and ectopic thyroid or parathyroid lesions (15). In this patient, the heterogeneous CT appearance with soft-tissue, fluid, calcified, and fat-density components supported a presumptive diagnosis of mature teratoma, which was subsequently confirmed by pathology; however, anesthetic risk stratification was driven primarily by the degree of tracheobronchial compression, pericardial effusion, and cardiopulmonary reserve rather than by the histologic diagnosis alone (16).

### Risk assessment and anesthetic plan

The main preoperative concern was the discordance between the patient’s limited symptoms and substantial imaging abnormalities. The coexistence of tracheal compression, left main bronchial narrowing, pericardial effusion, and class II obesity was considered sufficient to classify the patient as high risk for the induction of general anesthesia, despite his ability to tolerate the supine position (1–4, 7).

The plan was to maintain spontaneous ventilation until a secure airway and effective ventilation had been confirmed. A 37 Fr left DLT was selected because right thoracotomy and possible left-lung isolation were anticipated. Before entering the operating room, the team agreed on sequential backup strategies: (i) withdraw the DLT into the main airway and use both lumens for bilateral ventilation if bronchial positioning failed, (ii) exchange to a single-lumen tube (SLT) over an airway exchange catheter if ventilation through the DLT was inadequate, (iii) perform rigid bronchoscopy for severe airway obstruction, and (iv) initiate emergency extracorporeal membrane oxygenation (ECMO) if cardiorespiratory collapse could not otherwise be stabilized.

### Airway management and intraoperative course

No premedication was administered. The patient was placed in the semi-recumbent position. After standard monitoring and placement of a left radial arterial line, multilevel topical anesthesia was performed according to awake tracheal intubation principles with oropharyngeal tetracaine, lidocaine spray applied to the tongue base and epiglottic region, a transcricothyroid injection of 2% lidocaine, and tetracaine gel on the distal DLT (total lidocaine 130 mg, 1.14 mg/kg actual body weight) (10). No signs of local anesthetic systemic toxicity were observed. With the patient fully awake and breathing spontaneously, video laryngoscopy revealed a Cormack–Lehane grade I view. A 37 Fr left DLT passed through the glottis without difficulty. Fiberoptic bronchoscopy performed through the bronchial lumen revealed a flattened trachea, a displaced carina, and a slit-like left main bronchial orifice. The bronchial tip encountered definite resistance at the entrance to the left main bronchus and could not be advanced safely. The DLT was withdrawn into the main airway, with its tip approximately 2–3 cm above the carina (depth 24 cm at the teeth). The tracheal cuff was inflated, and the bronchial cuff was left deflated. Both lumens were connected for bilateral ventilation, which was confirmed by fiberoptic bronchoscopy, bilateral chest movement, and continuous end-tidal carbon dioxide waveform. Oxygen saturation remained at or above 99%, and spontaneous breathing was preserved throughout the airway instrumentation.

After bilateral ventilation had been confirmed, general anesthesia was induced with propofol 40 mg, fentanyl 0.1 mg, and vecuronium 4 mg. Hemodynamics remained stable (blood pressure approximately 115/65 mmHg, heart rate approximately 75 bpm). A right internal jugular central venous catheter was placed. Lung-protective bilateral ventilation was initiated at a tidal volume of 6–7 mL/kg ideal body weight with a positive end-expiratory pressure of 5 cmH₂O. During the closed-chest phase, arterial blood gas analysis showed a pH of 7.289, PaCO₂ of 52.7 mmHg, and PaO₂ of 250.4 mmHg, consistent with permissive hypercapnia and preserved oxygenation. After thoracotomy, PaCO₂ improved to 46.1 mmHg without any change in ventilator settings. Hemodynamics remained stable throughout the procedure without vasopressor support.

### Surgical procedure, diagnostic assessment, and outcome

A right fourth-intercostal anterolateral thoracotomy was performed. An intact encapsulated mass was found compressing the trachea and left main bronchus posteriorly. Adhesions to the mediastinal pleura and pericardium were present, but a dissection plane was identifiable. Complete *en bloc* resection was achieved in 2 h 35 min. Estimated blood loss was 200 mL, and no blood transfusion was required. Pathologic examination confirmed mature teratoma.

Approximately 15 min after surgery, the patient was awake, had spontaneous tidal volumes exceeding 500 mL, and maintained an oxygen saturation of 100%. The DLT was removed without complications, and the patient was transferred to the pediatric intensive care unit. Postoperatively, increased pericardial effusion, left pleural effusion, and left lower lobe atelectasis were managed conservatively with chest drainage and supportive care. The patient was discharged on postoperative day 23. At the 3-month follow-up, chest CT showed no recurrence, resolution of pericardial effusion, and full bilateral lung re-expansion.

## Discussion

This case demonstrates that limited symptoms do not necessarily indicate low anesthetic risk in adolescents with anterior mediastinal masses. The absence of stridor, orthopnea, positional desaturation, or supine intolerance contrasts with marked tracheobronchial compression and pericardial effusion on imaging. Previous pediatric and adult series have emphasized the importance of symptoms, airway compression, bronchial involvement, great-vessel compression, and pericardial effusion in perioperative risk assessment (1–7). Béchard et al. (3) identified pericardial effusion as the only independent predictor of intraoperative complications in the largest adult case series to date; however, their cohort was not stratified by body mass index. Anghelescu et al. (6) found that clinical symptoms and imaging findings predicted anesthetic complications in children with malignant mediastinal masses; however, obesity was not examined as a potential confounding variable.

Obesity may have contributed to the difficulty in interpreting symptoms in this case, although a causal relationship cannot be established from a single observation. Severe obesity is associated with reduced functional residual capacity, reduced expiratory reserve volume, increased baseline airway resistance, and adoption of a rapid shallow breathing pattern (8, 9). These changes may limit exertional activity and reduce the opportunity for patients to notice dyspnea while also shortening the time available for rescue during apnea or airway obstruction. In obese adolescents with anterior mediastinal masses, imaging-based quantification of the trachea, main bronchi, and cardiovascular structures may therefore deserve particular consideration in risk stratification, even when symptoms are mild. Published thresholds, such as tracheal compression exceeding 50% (1, 7, 17), provide a starting point, although whether obesity should lower the threshold for escalating anesthetic precautions remains an open question.

**Figure 2:**
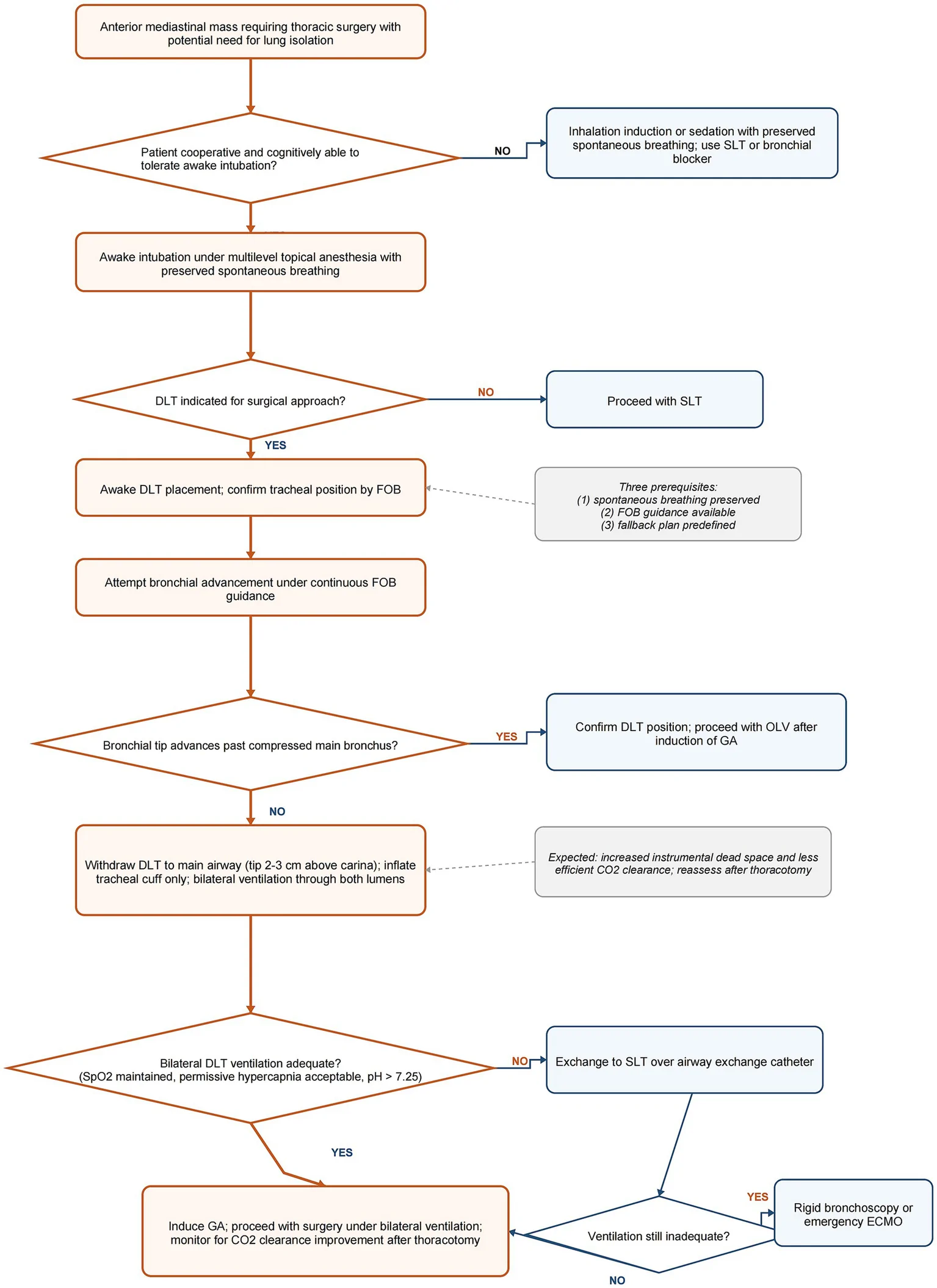
Proposed intraoperative decision pathway for DLT placement and ventilation strategy conversion in an anterior mediastinal mass with airway compression. The orange highlighted pathway represents the course taken in the present case. DLT, double-lumen tube; FOB, fiberoptic bronchoscopy; GA, general anesthesia; OLV, one-lung ventilation; SLT, single-lumen tube; ECMO, extracorporeal membrane oxygenation.

The safety of the present approach rested on a single organizing principle: securing the airway before induction while preserving spontaneous ventilation. Awake intubation was chosen to avoid the simultaneous loss of airway tone and spontaneous breathing before a functional airway strategy had been established, which was consistent with difficult-airway principles that favor awake tracheal intubation when failure of rescue ventilation would carry serious consequences (10). Bronchial advancement was performed only under continuous fiberoptic visualization, was stopped as soon as firm resistance was encountered, and was backed by a fallback strategy that had been communicated to the entire team before induction. These preconditions determined whether the DLT attempt remained controlled or became exploratory; in the absence of any one of them (spontaneous breathing, fiberoptic guidance, or a predefined alternative), attempting bronchial positioning in a compressed airway would not have been justifiable (Figure 2).

The most distinctive decision was to retain the DLT in the main airway and ventilate bilaterally through both lumens after bronchial positioning failed rather than exchanging the tube. This was not equivalent to conventional DLT use for lung isolation; it was a temporary rescue configuration selected because the airway had already been secured, oxygenation was preserved, and tube exchange in the setting of a distorted carina, left main bronchial compression, obesity, and pericardial effusion was considered to be at higher risk. Some authors have cautioned against the use of DLT in patients with large mediastinal masses because the larger outer diameter and relative rigidity of the tube may make its insertion hazardous in a compressed or distorted airway (11). That concern relates to the risk of insertion rather than the physiology of ventilation through a DLT once it is already in place (see Table 1).

**Table 1:** Timeline of clinical course.

Time point	Event	Key data and decisions
4 months before admission	Symptom onset	Chest tightness and chest pain
1 month before admission	Symptom progression	Worsening cough; outside CT: anterior mediastinal mass
Admission	Risk workup	CT: 10.4 × 9.7 cm mass, tracheal and left main bronchus compression; echo: pericardial effusion 16 mm, LVEF 63%; no orthopnea, stridor, or positional desaturation
Surgery	Awake DLT intubation	Semi-recumbent; multilevel topical anesthesia; 37 Fr left DLT placed awake; spontaneous breathing preserved; SpO₂ ≥ 99%
	Bronchial positioning fails	FOB: slit-like left main bronchus; DLT withdrawn to main airway; bilateral ventilation through both lumens confirmed
	GA induction	Propofol/fentanyl/vecuronium; hemodynamically stable
	Closed-chest phase	ABG: pH 7.289, PaCO₂ 52.7, PaO₂ 250.4
	Thoracotomy and resection	PaCO₂ improved to 46.1 mmHg; en bloc resection; 2 h 35 min; EBL 200 mL
	Extubation	Awake, spontaneous VT > 500 mL, SpO₂ 100%; DLT removed; transferred to PICU
POD 1 to 3	Postoperative course	Pericardial effusion, left pleural effusion, LLL atelectasis; managed with drainage and supportive care
POD 23	Discharge	Clinically improved
3-month follow-up	Recovery confirmed	CT: no recurrence, effusions resolved, lungs fully expanded

ABG, arterial blood gas; DLT, double-lumen tube; EBL, estimated blood loss; FOB, fiberoptic bronchoscopy; GA, general anesthesia; LLL, left lower lobe; PICU, pediatric intensive care unit; POD, postoperative day; VT, tidal volume.

Using nominal circular-lumen geometry for a 37 Fr left DLT, with an approximate tracheal lumen inner diameter of 6.5 mm and bronchial lumen inner diameter of 5.5 mm, the combined geometric area of the two lumens is approximately 56.9 mm^2^, compared with approximately 50.3 mm^2^ for an 8.0-mm inner-diameter SLT. This comparison should not be interpreted as a direct estimate of airflow resistance because resistance is also influenced by tube length, curvature, connectors, bifurcation geometry, flow regime, and residual extrinsic airway compression. In this rescue configuration, the more clinically relevant disadvantage was likely increased instrumental dead space and less efficient CO₂ clearance rather than impaired oxygen delivery. This interpretation is consistent with the observed transient hypercapnia during the closed-chest phase, while oxygenation remained preserved. Because dead space, airway resistance, and respiratory mechanics were not directly measured, these observations should be regarded as physiologic inferences rather than measured proof.

The improvement in PaCO₂ from 52.7 to 46.1 mmHg after thoracotomy, without any change in ventilator settings, is consistent with reduced chest wall elastic recoil and partial relief of extrinsic bronchial compression during tumor mobilization. This pattern suggests that the period of greatest hypercapnia risk is the pre-thoracotomy phase and that tolerating permissive hypercapnia within accepted limits (target pH above 7.25) during this finite window may represent a reasonable trade-off against the hazards of tube exchange (17, 18). Lee et al. (13) described a related scenario in which a DLT was used as an airway stent to maintain patency of a compressed carina during surgery for an anterior mediastinal mass. Together with the present case, their report suggests that the role of DLT in mediastinal mass surgery may extend beyond conventional lung isolation to include airway stenting and, as observed here, temporary rescue bilateral ventilation.

The majority of published pediatric mediastinal mass cases employ inhalational induction with preserved spontaneous breathing or light sedation with dexmedetomidine (1, 4–6). Fully awake, sedation-free DLT placement in adolescents appears to be rarely described. The feasibility of this approach in the present case depended on the patient’s cognitive maturity, which permitted understanding and cooperation after preoperative counseling, as well as meticulous multilevel topical anesthesia. The outer diameter of a 37 Fr DLT is approximately 13.0 mm, approaching the expected tracheal internal diameter of a 13-year-old boy (approximately 13 to 14 mm), leaving minimal margin. Any coughing or laryngospasm at this size could cause glottic trauma, underscoring the importance of effective surface analgesia. This approach should not be extrapolated to younger or less cooperative children, for whom titrated sedation with preserved spontaneous ventilation remains more appropriate (1, 4).

Several limitations should be acknowledged. First, airway narrowing was assessed using routine CT images rather than quantified with dedicated airway analysis software or preoperative flow-volume loop testing, the latter of which is recommended by some guidelines for the functional assessment of intrathoracic obstruction (2, 7). Therefore, the degree of tracheal and left main bronchial compression could not be reported with the precision desirable for risk-stratification comparisons. Second, although the pattern of preserved oxygenation with transient hypercapnia was physiologically compatible with increased instrumental dead space and altered airflow resistance during bilateral DLT ventilation, dead space, airway resistance, and respiratory mechanics were not directly measured. Third, fiberoptic reassessment of the left main bronchus after tumor removal was not performed, which would have provided useful information regarding the immediate reversibility of bronchial compression. Therefore, these observations should be interpreted as hypothesis-generating and applicable only to carefully selected patients.

## Conclusion

In an obese adolescent with a giant anterior mediastinal teratoma, awake DLT intubation with preserved spontaneous breathing enabled airway control before induction. When bronchial positioning failed, temporary bilateral ventilation through both DLT lumens provided effective oxygenation with transient, physiologically explainable hypercapnia that improved after thoracotomy. This case supports a cautious, airway-first approach and suggests that temporary bilateral DLT ventilation may be considered as a rescue option in selected patients when airway exchange is judged to pose a greater risk than accepting short-term permissive hypercapnia.

## Patient and family perspective

The patient’s family was informed preoperatively about the need for awake intubation, the possibility of intraoperative conversion of the ventilation strategy, and the planned rescue options. After surgery and at follow-up, the family expressed satisfaction with the successful extubation, recovery, and absence of recurrence at 3 months.

## Data Availability

The raw data supporting the conclusions of this article will be made available by the authors, without undue reservation.
